# Molecular Detections of Hematoparasites in Tabanids (Diptera: Tabanidae) from Northeastern Thailand

**DOI:** 10.3390/pathogens14121208

**Published:** 2025-11-27

**Authors:** Bhuvadol Gomontean, Wannachai Wannasingha, Waraporn Jumpato, Ailada Nabkrathok, Chawanut Jaroenchaiwattanachote, Komgrit Wongpakam, Pairot Pramual

**Affiliations:** 1 Department of Biology, Faculty of Science, Mahasarakham University, Mahasarakham 44150, Thailand; bhuvadol.g@msu.ac.th (B.G.); waraporn.a2536@gmail.com (W.J.); ailada26736@gmail.com (A.N.); 2 Center of Excellence in Biodiversity Research, Mahasarakham University, Mahasarakham 44150, Thailand; wannachai.w@msu.ac.th (W.W.); chavanut.j@msu.ac.th (C.J.); 3 Walai Rukhavej Botanical Research Institute, Mahasarakham University, Mahasarakham 44150, Thailand; komwongpa@gmail.com

**Keywords:** *Tabanus*, *Chrysops*, horse fly, deer fly, *Trypanosoma*, *Anaplasma*

## Abstract

Tabanid flies (Diptera: Tabanidae) are important pests and potential vectors of various pathogens affecting humans and animals. However, information on their vectorial roles in Thailand remains limited. In this study, we employed molecular methods to screen for filarial nematodes and hemoparasites in four tabanid species collected from northeastern Thailand. A total of 246 specimens representing *Chrysops dispar* Fabricius, *Tabanus rubidus* Wiedemann, *T. megalops* Walker, and *T. striatus* Fabricius were examined. No filarial parasites were detected. However, three specimens—one from each *Tabanus* species—tested positive for *Trypanosoma*. Analysis of ITS1/5.8S rRNA/ITS2 sequences revealed that the detected *Trypanosoma* were most closely related to the *T. theileri* complex, showing >98% sequence similarity. Phylogenetic analyses further indicated that the *T. theileri* complex detected in this study clustered with *T. theileri* lineage II (TthII). Additionally, one specimen of *T. megalops* tested positive for *Anaplasma*, with 16S rDNA sequences showing 99.76% similarity to *A. platys*. To our knowledge, this is the first report of the *T. theileri* complex in *T. megalops*, *T. rubidus*, and *T. striatus*, suggesting that these species may serve as potential mechanical carriers of these pathogens.

## 1. Introduction

Hematophagous flies of the family Tabanidae comprise approximately 4455 species [1] distributed across all continents except Antarctica [2]. The family is divided into four subfamilies: *Chrysopsinae*, *Pangoniinae*, *Scepsidinae*, and *Tabaninae*. Many species of the genera *Chrysops* Meigen and *Tabanus* Linnaeus, belonging to the subfamilies *Chrysopsinae* and *Tabaninae,* respectively, are important pests and vectors of disease-causing agents affecting humans and other animals [3].

Tabanid flies are capable of transmitting a variety of pathogens either mechanically or biologically, although most transmission occurs mechanically [2,3]. Major pathogens known to be transmitted by tabanids include *Trypanosoma evansi* (the causative agent of surra), equine infectious anemia virus (the agent of equine infectious anemia), *Anaplasma marginale* (a rickettsia responsible for bovine anaplasmosis), and *Loa loa* (a filarial nematode causing loiasis in humans) [2,3]. In addition to their vectorial role, tabanids are also major pests of livestock. Their bites cause stress, blood loss, and irritation, leading to significant reductions in livestock productivity [3,4].

Identifying which tabanid species are capable of transmitting particular pathogens is critical for understanding disease epidemiology. In Thailand, approximately 80 species of tabanids have been recorded [5,6]. Only nine *Chrysops* species are currently known from Thailand [7], though additional species likely await discovery, as cryptic genetic diversity has been detected within some taxa [7,8,9]. Despite the widespread occurrence of tabanids and the high prevalence of pathogens in potential vertebrate hosts, knowledge of pathogen associations in Thai tabanids remains limited. For example, over 20% of cattle in Thailand have been reported to harbor the *Trypanosoma theileri* complex [10,11], while infections with Anaplasmataceae have been detected in 11% of beef cattle and 41% of water buffalo [11,12].

To date, only a few studies have investigated pathogens in tabanid vectors from Thailand. Molecular screening of tabanids from southern Thailand revealed the presence of *Anaplasma*, *Ehrlichia*, *Babesia*, and *Theileria*, although all were detected exclusively in *Tabanus megalops* Walker [13,14]. *Theileria* spp. have also been reported from unidentified *Tabanus* species in northeastern Thailand [15]. Furthermore, the *Trypanosoma theileri* complex was recently detected in the deer fly *Chrysops dispar* Fabricius from the same region [9].

In this study, we molecularly screened for filarial nematodes, blood protozoa (*Trypanosoma*, *Babesia*, and *Theileria*), and rickettsiae (*Anaplasma*) in four common tabanid species—*Tabanus striatus* Fabricius, *T. megalops* Walker, *T. rubidus* Wiedemann, and *Chrysops dispar* Fabricius—collected from cattle pens in northeastern Thailand. The findings of this study contribute to a better understanding of the potential roles of these tabanids in pathogen transmission.

## 2. Materials and Methods

### 2.1. Specimen Collection and Identification

**Figure 1:**
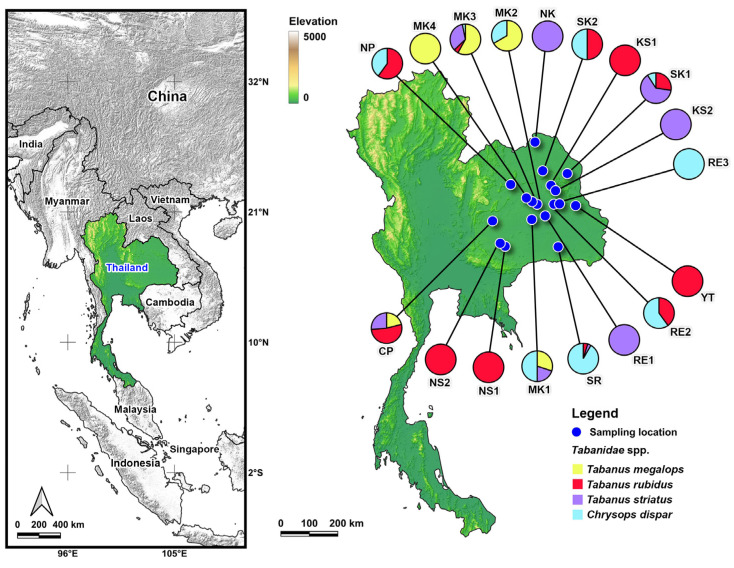
Map of collection sites of four tabanid species from northeastern Thailand. Colors represent species found at each sampling site. Details of each sampling site were provided in Table S1.

Tabanidae specimens were collected from the areas around the cattle pens using a sweep net. We also collected the flies by hand while they were attached to cattle. Specimens were collected early in the morning (06:00–08:00) and late in the afternoon (16:00–18:00), which are the times when cattle were in their shelters. Approximately 4–5 people participated in specimen collection. In total, we conducted 32 collections and obtained 246 specimens from 18 locations across 10 provinces in the northeastern region of Thailand (Table 1 and Table S1, Figure 1). The majority of collections (26 out of 32) were carried out during the rainy season (mid-May to October), when tabanid flies are highly abundant [16]. Specimens were preserved in 80% ethanol and stored at −20 °C in a freezer until investigation. Tabanid flies were identified using keys to Tabanidae in Thailand [5,6].

### 2.2. Molecular Detection of Pathogens

In total, 246 specimens of four tabanid species: *Chrysops dispar* (*n* = 100), *Tabanus rubidus* (*n* = 48), *T. megalops* (*n* = 48) and *T. striatus* (*n* = 50) were used for pathogen detection (Table 1). For each individual specimen, the head and thorax were homogenized and used for DNA extraction using the GF-1 Nucleic Acid DNA extraction kit (Vivantis Technologies Sdn. Bhd., Subang Jaya, Malaysia). We used a PCR approach to screen for three pathogen groups (filarial, blood protozoa (*Trypanosoma*, *Babesia* and *Theileria*) and rickettsia (*Anaplasma*)) in tabanid flies. For detection of filarial pathogens, the primers specifically amplified an approximately 450 bp fragment of the 12S rRNA (12SF, 5′-GTTCCAGAATAATCGGCTA-3′) and 12SR (5′-ATTGACGGATG(AG)TTTGTACC-3′) and the PCR reaction conditions described by Casiraghi et al. [17] were used. DNA of the filarial nematode extracted from *Culicoides* [18] was used as a positive control.

Trypanosomes were screened using the nested PCR method of Cox et al. [19]. The primers ITS1 (5′-GATTACGTCCCTGCCATTTG-3′) and ITS2 (5′-TTGTTCGCTATCGGTCTTCC-3′) [19] were used in the first round of PCR to amplify an approximately 1000 bp DNA fragment that contained the ITS1/5.8S/ITS2/LSU rRNA region. PCR products from the first round were used as templates for the second round using primers ITS3 (5′-GGAAGCAAAAGTCGTAACAAGG-3′) and ITS4 (5′-TGTTTTCTTTTCCTCCG CTG-3′) [19]. DNA of *Trypanosoma* detected in *C. dispar* [9] was used as positive. Because some specimens (*n* = 90) of *C. dispar* had already been screened for trypanosomes [9], only newly collected (*n* = 17) specimens were subjected to this protozoan detection. *Babesia* was screened using primers Ba103F(5′-CCAATCCTGACACAGGGAGGTAGTGACA-3′) and Ba721R (5′-CCCCAGAACCCAAAGACTTTGATTTCTCTCAAG-3′) that specifically amplified an approximately 619 bp fragment of the 18S rRNA gene [20]. The PCR reaction conditions followed those described by Kledmanee et al. [20]. The DNA of this parasite, isolated from cattle [21] was used as a positive control. *Theileria* was screened using the primers THEIFP (5′-TAGTGACAAGAAATAACAATACGGGGCT-3′) and THEIRP (5′-CAGCAGAAATTCAACTACGAGCTTTTTAACT-3′), which amplify a 188 bp fragment of the 18S rRNA gene, following the PCR conditions described in Sontigun et al. [13]. *Anaplasma* was screened using the primers AnaEhr16S_f (5′-AGAGTTTGATCMTGGYTCAGAA-3′) and Ana-Ehr16S_r (5′-GAGTTTGCCGGGACTTYTTC-3′) that specifically amplified a fragment (460–520 bp) of the 16S rDNA gene [20]. The PCR reaction conditions were as described by Abanda et al. [22]. *Anaplasma* DNA isolated from cattle [23] was used as a positive control. Nuclease-free water was used as a negative control for all PCR reactions.

PCR products were stained with Novel Juice (GeneDirex, Taoyuan, Taiwan, Republic of China) and were checked using 1% agarose gel electrophoresis. Successful amplifications were purified using the PureDireX PCR CleanUp & Gel Extraction kit (Bio-Helix, New Taipei City, Taiwan, Republic of China). Purified PCR products were sequenced at ATCG Company Limited (Thailand Science Park, Pathumthani, Thailand) in both directions using the same primers as for PCR.

### 2.3. Data Analysis

The sequences of pathogens detected in tabanid flies were checked for quality using the “Edit/View sequencer file” in MEGA X [24]. In this study, a total of seven specimens were positive (visible PCR product) for *Trypanosoma* but only three were successfully sequenced. Unsuccessful sequencing is possibly due to the amplification of non-specific products of PCR or co-infection of more than one parasite species. These sequences (848 bp) were deposited in NCBI GenBank (accession nos. PV346806-PV346808) and also provided as Supplement Materials (Data S2). To identify the trypanosomes detected in tabanid flies, the sequences were compared with those recorded in the NCBI GenBank using the Basic Local Alignment Search Tool (BLAST) (https://blast.ncbi.nlm.nih.gov/Blast.cgi) (accessed on 15 March 2025). To examine genetic relationships between the trypanosomes detected in tabanid flies from this study and those recorded in GenBank, the sequences with >95% identity based on NCBI BLAST search were retrieved and included in phylogenetic analyses. In addition, sequences of the *Trypanosoma theileri* detected in *Chrysops dispar* [9] for the specimens used in the present study to examine other pathogens were also included in the phylogenetic analyses. Two phylogenetic methods, maximum likelihood (ML) and neighbor joining (NJ) were used. The NJ analysis was performed in MEGA X [24]. The Kimura 2-parameter model was selected for NJ tree analysis, and the branch support was based on 1000 bootstrapping replications. The ML tree was calculated in IQ-TREE [25,26] web server version (http://iqtree.cibiv.univie.ac.at/) (accessed on 14 March 2025). The Bayesian information criterion was selected K3Pu + F + G4 as the best-fit model. Branch support was calculated using the ultrafast bootstrap setting with 1000 replications. The sequence of *Anaplasma* (415 bp) detected in this study (accession nos. PX285886) was compared with those recorded in NCBI GenBank using a BLAST search for identification.

## 3. Results and Discussion

**Figure 2:**
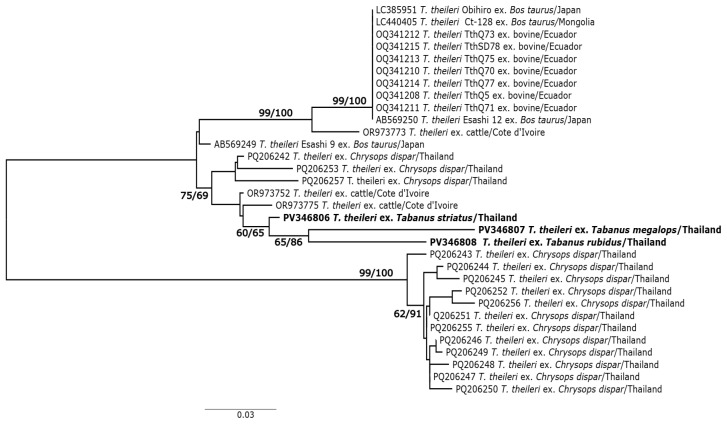
Maximum-likelihood phylogenetic tree inferred from the ITS1/5.8S rRNA/ITS2 sequences of the *Trypanosoma theileri* complex detected in *Tabanus* spp. (in bold) in this study, along with closely related sequences retrieved from the NCBI GenBank database. Bootstrap support values from maximum-likelihood (ML) and neighbor-joining (NJ) analyses are shown above or near the corresponding branches. For each node, the GenBank accession number is followed by the strain name, vertebrate host or invertebrate vector, and country of origin.

Molecular screening for filarial nematodes, blood protozoa (*Trypanosoma, Babesia*, and *Theileria*), and rickettsiae (*Anaplasma*) in *Chrysops dispar*, *Tabanus rubidus*, *T. megalops*, and *T. striatus* collected from northeastern Thailand detected two groups of pathogens: *Trypanosoma* and *Anaplasma*. *Trypanosoma* DNA was detected in three specimens—one each from *T. megalops*, *T. rubidus*, and *T. striatus*. The low rate of parasite detection in this study limited our ability to interpret the diversity of these parasites. However, comparison of the ITS1/5.8S rRNA/ITS2 sequences with those in GenBank indicated that all sequences belonged to the *Trypanosoma theileri* complex, showing greater than 98% similarity with 100% sequence coverage. Phylogenetic analyses revealed identical tree topologies using both maximum likelihood (ML) and neighbor-joining (NJ) methods; therefore, only the ML tree is presented (Figure 2). All three *T. theileri*-complex sequences from *Tabanus* species collected in northeastern Thailand clustered within *T. theileri* lineage II (TthII) [27].

Several *Trypanosoma* species are known to be transmitted by *Tabanus*, including *Trypanosoma evansi*, the causative agent of the important disease surra [2,3]. The *T. theileri* complex, which includes globally distributed species, has traditionally been regarded as nonpathogenic. However, *T. theileri* infections may become chronic and potentially pathogenic in animals with compromised immunity (e.g., newborn or pregnant cows) or those under high physical or nutritional stress [10]. *T. theileri* can be transmitted both mechanically and biologically by numerous *Tabanus* species [2,3], and it has been detected in diverse horse fly species from Southeast Asia [28], East Asia [29], West Africa [30], Europe [31], and South America [32].

Although the *T. theileri* complex has been reported at high detection rates (>20%) in cattle from Thailand [10,11], little is known about its potential vector species. The only prior report of a possible vector in Thailand involved the deer fly *Chrysops dispar* [9]. Both genetic lineages of *T. theileri* (TthI and TthII) were detected in *C. dispar*, which was also screened for other pathogens in this study. The detection of DNA of TthII in three *Tabanus* species (*T. rubidus, T. megalops,* and *T. striatus*) suggests that these common horse flies [16], along with *C. dispar*, may serve as potential mechanical carriers of the *T. theileri* complex in Thailand.

*Anaplasma* DNA was detected in one specimen of *T. megalops*. BLAST analysis revealed that the 16S rDNA sequence from this specimen showed high similarity (99.76%, 100% coverage) to *Anaplasma platys* isolated from domestic sheep in Kenya (accession no. KU569704) [33]. *Anaplasma* is a pathogenic rickettsia transmitted biologically by ticks and mechanically by tabanids. In cattle, *Anaplasma* infection causes anaplasmosis, leading to anemia, fever, weight loss, and potentially death [2]. Although tabanids are not considered primary vectors, several studies have reported the presence of *Anaplasma* spp. in various tabanid species [34,35,36,37]. The detection rate of *Anaplasma* in this study (1 of 246 individuals; 0.4%) was considerably lower than that observed in vertebrate hosts in Thailand, where infection rates of 41% in buffalo [12] and 11.6% in beef cattle [11] have been reported. Molecular detection in the principal vectors (ticks) revealed a higher detection rate (22%) [38] than in tabanids. A comparatively higher detection rate (8.6%) of *Anaplasma* was previously reported in *T. megalops* from southern Thailand [13]. Our findings provide an additional record of *Anaplasma* infection in this species from northeastern Thailand.

In summary, we detected DNA of two parasite groups—*Trypanosoma* and *Anaplasma*—in three tabanid species (*T. rubidus*, *T. megalops*, and *T. striatus*) from northeastern Thailand. Although the presence of parasite DNA in these flies does not confirm their ability to biologically transmit the pathogens, tabanids are known to transmit pathogens mechanically [2,3]. Our findings indicate that the common Thai horse flies *T. rubidus*, *T. megalops*, and *T. striatus* are potential mechanical carriers of both the *Trypanosoma theileri* complex and *Anaplasma* spp. While the detection rate of these hemopathogens in tabanids was relatively low compared to that in their primary vectors (ticks), the wide distribution and long-distance dispersal capacity of tabanid flies may contribute to the broader geographic spread of these pathogens and may facilitate disease outbreaks across regions. Further research using larger sample sizes of tabanid flies will be valuable for determining the diversity of parasites potentially transmitted by these insects.

## Figures and Tables

**Table 1:** Number of specimens of *Chrysops dispar*, *Tabanus megalops*, *T. rubidus* and *T. striatus* in 10 provinces from northeastern Thailand used for molecular detections of pathogens. Details of sampling sites in each province were provided in Table S1.

Province	Species	N
Chaiyaphum	*T. megalops*	4
	*T. rubidus*	10
	*T. striatus*	5
Kalasin	*T. rubidus*	10
	*T. striatus*	4
Maha Sarakham	*T. megalops*	44
	*T. striatus*	23
	*C. dispar*	8
Nong Khai	*T. striatus*	3
Nong Bua Lamphu	*T. rubidus*	3
	*C. dispar*	2
Nakhon Ratchasima	*T. rubidus*	5
Roi Et	*T. striatus*	10
	*T. rubidus*	6
	*C. dispar*	12
Sisaket	*T. rubidus*	4
	*T. striatus*	3
	*C. dispar*	77
Sakon Nakhon	*T. rubidus*	6
	*T. striatus*	7
	*C. dispar*	4
Yasothon	*T. rubidus*	5

## Data Availability

The data generated during the study have already been reported in the manuscript.

## Supplementary Materials

The following supporting information can be downloaded at https://www.mdpi.com/article/10.3390/pathogens14121208/s1, Table S1. Sampling locations of *Chrysops dispar*, *Tabanus megalops*, *T*. *rubidus* and *T*. *striatus* from northeastern Thailand for molecular detections of pathogens. Data S2.
