# Supplementary material for: Molecular Detections of Hematoparasites in Tabanids (Diptera: Tabanidae) from Northeastern Thailand
Source: Pathogens. 2025 Nov 27;14(12):1208. doi: 10.3390/pathogens14121208 (PMC12736051; doi:10.3390/pathogens14121208)
Supplement: Supplementary file 1 [file pathogens-14-01208-s001.zip › Table S1.pdf]

Table 1. Sampling locations of *Chrysops dispar*, *Tabanus megalops*, *T. rubidus* and *T. striatus* from northeastern Thailand for molecular detections of pathogens.

Location (code)	Coordinate	Elevation (m)	Species	N	Collection date
Chatturat, Chaiyaphum (CP)	15.650028 N / 101.983547 E	191	*T. megalops*	4	22 Dec 2023
			T. rubidus	10	28 Oct 2023
			T. striatus	5	28 Oct 2023, 22 Dec 2023, 23 Feb 2024
Somdet, Kalasin (KS1)	16.775222 N / 103.834778 E	208	*T. rubidus*	10	21 Oct 2023
Huai Phueng, Kalasin (KS2)	16.754056 N / 103.850306 E	210	*T. striatus*	4	9 Dec 2023
Na Dun, Maha Sarakham (MK1)	15.696722 N / 103.225694 E	157	*T. megalops*	3	9 Sep 2023
			T. striatus	2	5 Sep 2023
			C. dispar	5	5 Sep 2023, 24 Aug 2024
Mueang Maha Sarakham, Maha Sarakham (MK2)	16.217917 N / 103.334333 E	145	*T. megalops*	2	2 Sep 2023
			C. dispar	1	26 May 2024
Kantharawichai, Maha Sarakham (MK3)	16.260056 N / 103.239139 E	144	*T. megalops*	36	29 Oct – 13 Dec 2023
			T. rubidus	3	29 Oct – 11 Nov 2023
			T. striatus	21	6 Oct – 13 Dec 2023
			C. dispar	2	
Chiang Yuen, Maha Sarakham (MK4)	16.379167 N / 103.054333 E	164	*T. megalops*	3	31 Aug 2023
Rattanawapi, Nong Khai (NK)	18.148606 N / 103.328608 E	182	*T. striatus*	3	28 May 2022
Non Sang, Nong Bua Lamphu (NP)	16.805264 N / 102.558381 E	190	*T. rubidus*	3	23 Sep 2023
			C. dispar	2	
Chaloem Phra Kiat, Nakhon Ratchasima (NS1)	14.924818 N / 102.252981 E	197	*T. rubidus*	3	10 Aug 2024
Mueang, Nakhon Ratchasima (NS2)	14.942953 N / 102.225903 E	177	*T. rubidus*	2	10 Aug 2024
Chaturaphak Phiman, Roi Et (RE1)	15.810658 N / 103.650389 E	147	*T. striatus*	5	2 Sep 2023

Location (code)	Coordinate	Elevation (m)	Species	N	Collection date
Selaphum, Roi Et (RE2)	16.181861 N / 103.927278 E	145	*T. rubidus*	2	9 Sep 2023
			C. dispar	3	
Phon Thong, Roi Et (RE3)	16.181510 N / 103.937225 E	140	*C. dispar*	3	12 Sep 2023
Prang Ku, Sisaket (SR)	14.830383 N / 104.060561 E	147	*T. rubidus*	4	26 Aug 2023
			T. striatus	3	
			C. dispar	77	
Phon Na Kaeo, Sakon Nakhon (SK1)	17.149833 N / 104.350667 E	169	*T. rubidus*	3	28 Nov 2023
			T. striatus	7	22 Oct 2023, 28 Nov 2023
			C. dispar	1	
Waritchaphum, Sakon Nakhon (SK2)	17.238843 N / 103.573124 E	198	*T. rubidus*	3	6 Oct 2023
			C. dispar	3	
Loeng Nok Tha, Yasothon (YT)	16.136194 N / 104.615028 E	159	*T. rubidus*	5	9 Sep 2023
